# Another Important Lesson from Military Surgery?

**DOI:** 10.1016/j.ebiom.2015.08.005

**Published:** 2015-08-05

**Authors:** John W. Harmon

**Affiliations:** Department of Surgery and the Hendrix Burn/Wound Laboratory, Johns Hopkins University, Baltimore, MD, United States

Knowing when a wound can safely be closed is an iconic problem for surgery. When an extremity wound is closed too soon, with resulting infection requiring further debridement, an amputation can result. Or a minor amputation can be converted into a major amputation. Patient welfare is on the line, and expenses are considerable in these situations.

The novel work (Forsberg et al. 2015), published in this issue of EBioMedicine, utilized complex mathematical modeling using machine learning techniques to convert non-predictive individual markers into a useful pool construct with both high sensitivity and high specificity for predicting wound closure. This work needs to be extended to a larger pool of patients. It needs to be tested prospectively. It needs to be assessed in both military and civilian settings. Equipment needs to be designed to perform the multiple assays that are required. Real time computerized analysis of the data needs to be incorporated into the system so that predictive assessments can be available in a timely manner when they can be used to plan patient care.

Some would argue that this approach could not be useful in a military setting. It is true that as many as 10 measurements of serum and exudate markers may be necessary, and although computer assistance is required to process the data to produce a predictive assessment, it is conceivable that a device could be developed to make this approach clinically relevant for military casualties. These wounds require surgical debridement every few days. The collection of serum and wound exudate could be carried out at the time of debridement. Even in the military setting the debridement will be done in a surgical setting where these kinds of analyses could be carried out. These debridements would rarely be done in the chaos of the battlefield itself. With the right equipment this type of analysis could be performed for military casualties.

Imagining the use of this approach for civilian casualties is less problematic. And this approach would have a very high beneficial impact in the civilian setting where severe trauma is unfortunately an increasing problem.

If the approach explored in this paper reaches the clinic, this paper will join the ranks of the major medical advances from military medicine.

## Disclosure

I declare that I have no conflict of interest.
